# The significance and potential of functional food ingredients for control appetite and food intake

**DOI:** 10.1002/fsn3.2783

**Published:** 2022-02-18

**Authors:** Mina Esmaeili, Marjan Ajami, Meisam Barati, Fardin Javanmardi, Anahita Houshiarrad, Amin Mousavi Khaneghah

**Affiliations:** ^1^ Department of Nutrition Research National Nutrition and Food Technology Research Institute/School of Nutrition Sciences and Food Technology Shahid Beheshti University of Medical Sciences Tehran Iran; ^2^ Department of Food and Nutrition Policy and Planning National Nutrition and Food Technology Research Institute School of Nutrition Sciences and Food Technology Shahid Beheshti University of Medical Sciences Tehran Iran; ^3^ Department of Cellular and Molecular Nutrition Faculty of Nutrition and Food Technology National Nutrition and Food Technology Research Institute Shahid Beheshti University of Medical Sciences Tehran Iran; ^4^ Department of Food Science and Technology National Nutrition and Food Technology Research Institute/School of Nutrition Sciences and Food Technology Shahid Beheshti University of Medical Sciences Tehran Iran; ^5^ Department of Food Science and Nutrition Faculty of Food Engineering University of Campinas (UNICAMP) Campinas Brazil

**Keywords:** appetite, functional food, intake regulation, satiation, satiety

## Abstract

Dramatically rising global levels of obesity have raised consumers’ commercial and public health interest in foods that may help control appetite and weight. The satiety cascade consists of sensory, cognitive, physical, and hormonal events following food intake, preventing overeating, and the desire to eat for a long time. Functional foods can be one of the most influential factors in reducing appetite as long as effective ingredients, such as fiber and protein, are used to design these products. Also, functional foods should be designed to reduce appetite at different levels of oral processing, stomach, small intestine, and large intestine by various mechanisms. Therefore, the satiety power of functional foods depends on the type of ingredients and their amount. Because each compound has a different mechanism of action, it is recommended to use different compounds to influence satiety in functional foods.

## Graphical abstract

Functional foods can be one of the most influential factors in reducing appetite as long as effective ingredients, such as fiber and protein, are used to design these products. Also, functional foods should be designed to reduce appetite at different levels of oral processing, stomach, small intestine, and large intestine by various mechanisms. Therefore, the satiety power of functional food depends on the type of ingredients and their amount. Because each compound has a different mechanism of action, it is recommended to use different compounds to influence satiety in functional foods.

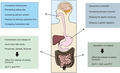

## 1 INTRODUCTION

Nowadays, talking about satiety and controlling appetite seems more necessary than ever. It can be imagined that a high percentage of the world population is overweight, while some face inadequate food and unbearable hunger. In 2016, according to the World Health Organization (WHO), it was estimated that 1.9 billion adults over 18 years of age were overweight in the world (39% of men and 40% of women). More than 650 million people were obese (11% of men and 15% of women) (WHO, 2016). These statistics show that obesity and overweight have become a global problem. Obesity is a risk factor for many health problems, including high blood pressure, high cholesterol, diabetes, cardiovascular disease, musculoskeletal diseases, and some types of cancers. Mortality also gradually increases as weight exceeds the threshold level (Bendor et al., 2020; Bray, 2021; Wilding & Jacob, 2021).

Various factors significantly contribute to the increase in obesity and overweight. The availability of processed, energy‐dense foods and beverages, especially those consumed between meals (Tey et al., 2018), and the increased serving size of products due to consumer demand have created an unfavorable atmosphere to increase food intake (Vien et al., 2019). An increase in food intake (calories consumed) and reduction in energy expenditure are considered important factors in increasing obesity and overweight (Williams et al., 2015). However, the increasing number of overweight and obese people globally showed that controlling food intake is complex, and many people cannot quickly reduce their food intake (Hu et al., 2020).

Food factories are constantly attempting to launch products that consumers are more willing to consume. Their purpose is to offer products leading to a greater appetite to consume. Therefore, food manufacturers are always considered part of the high prevalence of obesity and overweight problems. However, they can be considered a part of the solution (Hetherington et al., 2013). Hence, many food factories worldwide are revising their product formulations to produce products that can reduce appetite and food intake, especially in obese and overweight people (Hunter et al., 2019).

Despite significant scientific advances in understanding the relationship between specific nutrients and appetite control, there are still not many products on the market that effectively reduce appetite. Therefore, in this review, we investigated the relationship between the most effective nutrients with reduced appetite in different stages of oral processing, stomach, small intestine, and large intestine.

## 2 FUNCTIONAL FOODS CLAIMING TO REDUCE APPETITE

Efficacy, feasibility, and effect size are three critical parameters in offering functional foods to reduce appetite (Blundell, 2010). Some compounds indirectly influence appetite. Also, each product must be feasible in terms of production, process, and storage equipment. In addition, the amount of effect in each compound or sum of compounds on appetite is important in the design of such products (de Boer et al., 2016). In Canada and the European Union, functional food claims are divided into claims regarding reducing disease risk or treatment, claims related to performance, and general health claims. Functional food claiming to reduce appetite is placed into products with performance‐related claims. In this way, they influence the body's function or feeling of appetite and modulate it (Health, 2012). Such products are often abused by most manufacturers and cause misunderstandings among consumers. Reliable and scientific studies must substantiate any claim of reduction of appetite. Any claim of weight loss that may result from the use of appetite suppressants should be substantiated in long‐term human studies (J. Blundell, 2010). In addition, any claim to reduce appetite should be made comparatively. Hence, two groups of control and intervention must be selected, the amount of appetite reduction in them is examined, and confounding factors should be considered (López‐Nicolás et al., 2016). Usually, in the market, terms such as “loss of appetite,” “weight loss,” “feeling full for a long time,” “decreased appetite for a long time,” “appetite modulator,” “increased satiety,” and “delaying hunger” are used on labels of such products.

Food products that reduce appetite usually have less flavor and taste than other products because palatable foods are usually high‐calorie (Spence et al., 2016). Appetite increases by increasing the palatability of the food, followed by an increase in food intake, and there is a direct relationship between the palatability of the food and the increase in food intake and overeating (McCrickerd & Forde, 2016). Therefore, in the design and formulation of functional foods to reduce appetite, the purpose is to produce low‐calorie products, such as fibers, which influence the texture and taste of the product and reduce its palatability (Deighton et al., 2016). Also, such products usually have a high protein content, increasing the amount of protein decreasing the product's flavor (Van Kleef et al., 2012).

In functional food claiming to regulate appetite, the product's palatability is decreased by increasing the health effect. Therefore, consumers’ desire to consume such products decreases (Yuan et al., 2014). The manufacturer is faced with two ways; does it increase the beneficial effect of the product at the expense of losing the product's palatability? Or, does it reduce the beneficial effect of the product and increase the palatability of the product? (Anguah et al., 2017). The answer to these questions can be found in the study by Arguin et al. (2012). Arguin et al. conducted a study on 93 volunteers aged between 4 and 12 years old and evaluated one type of palatable and high‐energy lunch with seven healthy and low‐energy lunch types regarding food satisfaction and acceptance. The results of acceptability did not differ significantly between the samples. These results showed that being as healthy as palatability will be attractive to food, and if a functional product has beneficial properties and these properties are perceptible to the consumer, then claiming to be healthy will be a cover for the palatability of the food and the consumer tends to consume it (Blundell & Bellisle, 2013).

Functional foods are usually more expensive than regular food products. Functional dairy products are 30%–50% more expensive. At the same time, other functional foods are up to 500% more expensive (Bigliardi & Galati, 2013; Menrad, 2003). Usually, in most products designed and produced to decrease appetite, proteins and fibers are among the significant components of the product, and the price of fibers and proteins is usually much higher than that of other ingredients used in the food industry. Also, such products may reduce a part of an individual's nutrient intake during the day. To prevent malnutrition, a complex of vitamins and minerals will be added to such products, increasing the cost of these products. Therefore, it can be admitted that the target community of most functional food includes the population or groups with high social and economic status, which is considered one of the weaknesses of this type of product (WHO, 2014).

## 3 SATIETY MECHANISM

**FIGURE 1:**
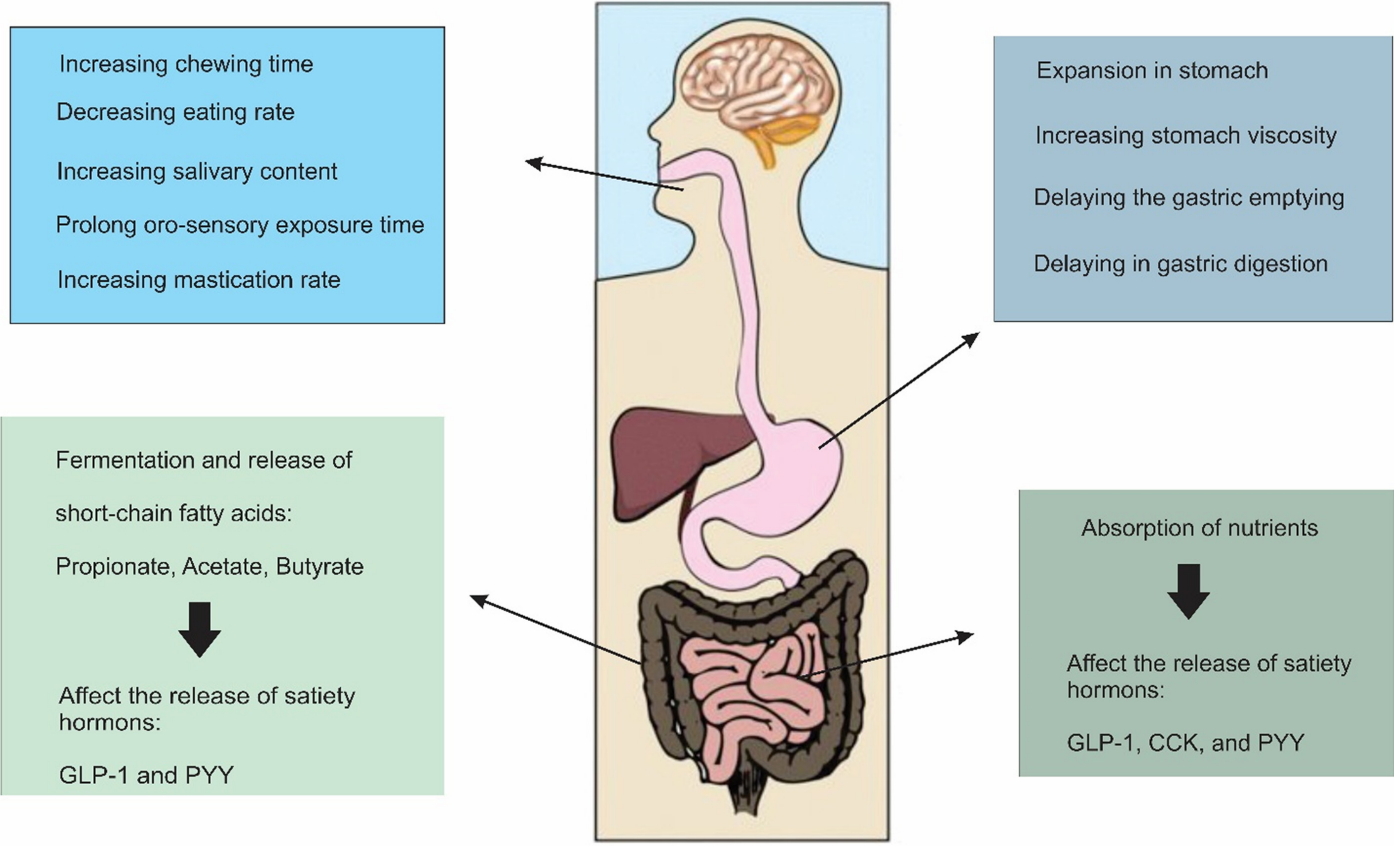
Potential actions of appetite control foods and ingredients. CCK, Cholecystokinin; GLP‐1, Glucagon‐like peptide 1; PYY, Peptide

Satiety mechanisms occur throughout the gastrointestinal tract, including chewing and saliva secretion, nutrient absorption time, gastric emptying time, gastric distension, secretion of intestinal hormones, time of food transfer in the small intestine, and fermentation in the large intestine (De Graaf et al., 2004) (Figure 1). The hypothalamus controls the appetite and is directly related to environmental compounds secreted from the gastrointestinal tract associated with satiety and food intake (Heisler & Lam, 2017). Many hormonal compounds and bioactive peptides are expressed or secreted in the gut, regulating appetite, food intake, and digestion through chemical and mechanical stimuli (Marić et al., 2014). Gut hormones playing an important role in regulating appetite include glucagon‐like peptide 1 (GLP‐1), glucagon‐like peptide 2 (GLP‐2), peptide YY (PPY), pancreatic polypeptide, oxyntomodulin, ghrelin, cholecystokinin (CCK), glucagon, and amylin (Miller, 2019). Ghrelin and GLP‐2 stimulate appetite and increase food intake, while other hormones alleviate appetite and reduce food intake (Perry & Wang, 2012). GLP‐1, PPY, and CCK are the most important hormones (Zanchi et al., 2017). GLP‐1 secretion–vagal afferent axis and meal sequence appear to play essential roles in controlling appetite (Iwasaki et al., 2018).

## 4 FACTORS INFLUENCING SATIETY

**FIGURE 2:**
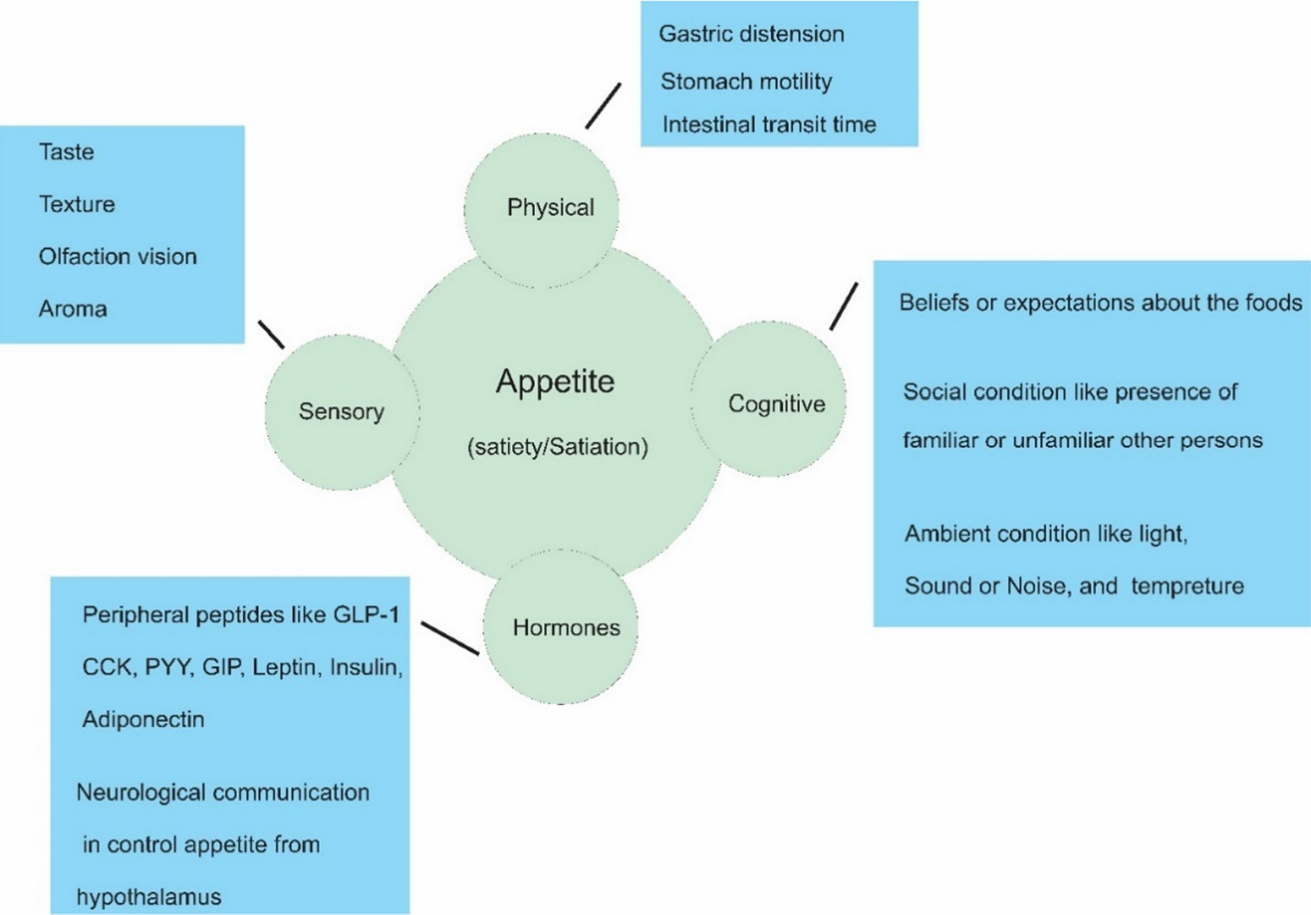
Internal and external factors affecting appetite. CCK, Cholecystokinin; GIP, Gastric inhibitory polypeptide; GLP‐1, Glucagon‐like peptide 1; PYY, Peptide YY

Various signals are produced during the consumption of food and beverages and subsequent events, including sensory, cognitive, hormonal, and metabolic signals, preventing more food intake (Figure 2). As shown in Figure 2, several factors can influence appetite, leading to appetite control being extremely difficult in practice (J. E. Blundell & Bellisle, 2013). Two terms with different meanings have been applied in the literature for appetite. The first is the concept of satiation, which refers to the variables that lead to stopping eating and giving up a meal. This concept shows that appetite is influenced not only by macronutrients including proteins, fats, and carbohydrates, but also by non‐nutritional variables, such as dietary fiber, taste, aroma, visual appearance of food, food texture (solid, semisolid, and liquid), and mouthfeel of food, which are influential in satiation sensory (Bellisle & Blundell, 2013). The next concept is satiety, referred to as having no desire to eat until the next meal, and includes four factors: sensory, cognitive, pre‐absorptive, and postabsorptive (Yeomans, 2020). However, it is interesting to note that these factors, which cause satiety and satiation for a long time, can influence the amount of food consumed in the next meal (Bellisle & Blundell, 2013).

**FIGURE 3:**
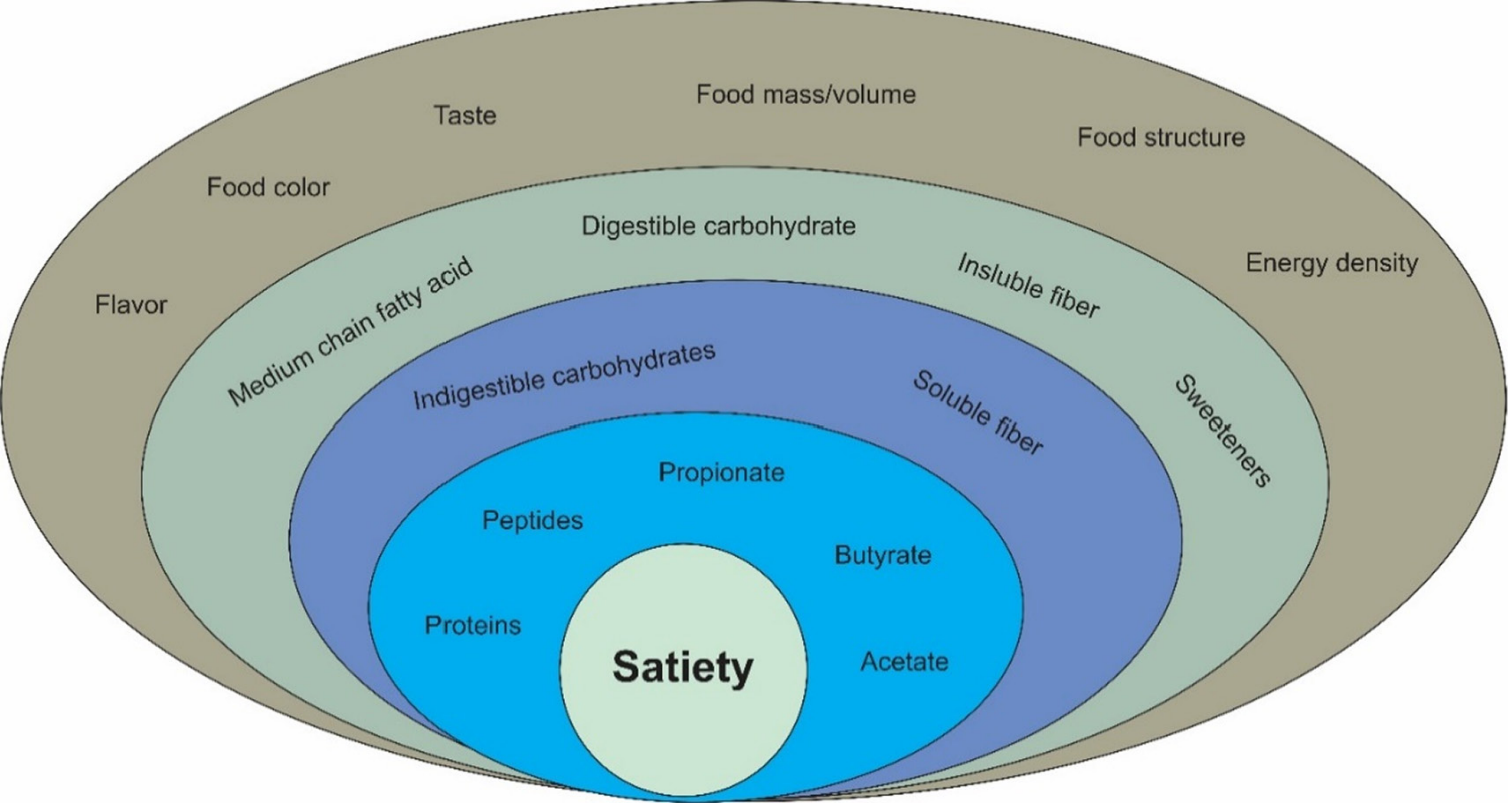
The effect of some food compounds on satiety according to the importance of their efficacy

The satiety process will begin with sensory properties of food, cognitive properties, and immediate responses of the stomach. Food palatability, related to food sensory factors, such as odor, taste, color, and texture, is the first step in releasing satiety signals (Chambers, 2016). Also, cognitive features, such as beliefs, voluntary control of food intake, eating alone or with friends and family, temperature, lighting, and noise of eating place, can be practical in the amount of food intake during meals or between meals (Yeomans, 2017). Finally, while digesting and absorbing food, some hormones are released, playing a role in satiety. This stage can be considered the most influential in controlling appetite (Booth & Nouwen, 2010) (Figure 3).

## 5 ORAL PROCESSING

Oral processing includes chewing of food, the activity of the jaw muscles, the number of chews, chewing time, the volume of bites in the mouth, and the time required to swallow (Mattes & Considine, 2013). Increasing or decreasing these factors can influence appetite. Stribiţcaia et al. (2020), in a systematic and meta‐analysis study on the results of 23 studies, showed that food texture manipulations, such as lubricant reduction, an increase in viscosity, chewiness, hardness, and product's elasticity, could have a positive effect on reducing appetite (Stribiţcaia et al., 2020). The eating rate of food in the mouth increases by increasing chewiness, elasticity, resilience, cohesiveness, and hardness, which causes food to be mixed with more saliva, and as a result, the volume of food in the gastric cavity increases and creates a feeling of fullness in the gastric, eventually leading to a feeling of satiety (McCrickerd et al., 2017). Also, increasing chewing time causes jaw muscles to be more involved, and the body has more opportunities to send satiety signals to the hypothalamus (Hetherington & Regan, 2011).

Drinks and foods consumed quickly and having a short processing time in the mouth would cause overconsumption, which is related to the inability to produce or reduce sensory signaling levels during eating. It ultimately leads to limited cephalic phase responses and delayed onset of satiety (Wanders et al., 2011). During food chewing in the mouth, it is hypothesized that perception of satiety through the mouth depends on the type of macro‐ and micro molecules in the food, texture of the food and its transmission during chewing, physiological activities required during bolus processing to form a bolus, and time of oral processing (Campbell et al., 2017). Studies have shown that the increase in food hardness can significantly increase muscle activity during chewing, chewing time, the number of chews to prepare food for swallowing, and range of motion of the jaw in the vertical and internal lateral plates (Cakir et al., 2012; Koç et al., 2014). In addition to food hardness, other food parameters, such as cohesiveness, elasticity, chewiness, water content, and fat content, play an important role in oral processing (Ishihara et al., 2011). Accordingly, it is necessary to use compounds like fibers to produce these characteristics. Water‐soluble fibers have a high water absorption power, making high‐fiber products with high hardness and cohesiveness. Fibers are usually found as natural fibers in the structure of products, such as whole grains and legumes, or as pure fiber in the formulation of food products. Certainly, water‐soluble fibers used purely in product formulations have a more significant effect on the textural parameters of the product (Yang et al., 2017). Fibers with high water absorption capacity like konjac gum, beta‐glucan, alginate, guar gum, and pectin can create high viscosity in the formulation and the gastrointestinal tract (Clark & Slavin, 2013).

The amount of water and oil in the product are important factors for reducing appetite (Halford & Harrold, 2012). Usually, the time between the first bite and swallowing of the food depends on the breakdown of the structure and increase in lubrication of each food in chewing conditions and fundamental characteristics of food (microstructure, physical and chemical properties) (Bolhuis et al., 2014). Therefore, the first bite to swallowing duration is reduced by increasing the product's water content. Thus, less opportunity would stimulate satiety signals (Chen, 2009). Also, products with high oil content require less saliva to lubricate the bite than products with low oil content. Therefore, such products reach swallowing conditions sooner (Andrade et al., 2008; Higgs & Jones, 2013).

Given the mentioned explanations, it is proved that products designed to reduce appetite and food intake have the most significant effect on satiation in the first stage (i.e., mouth). Thus, their texture parameters and sensory properties must be designed in such a way that the product spends most of its time in the mouth and engages muscles of the mouth as fully as possible so that the body has the opportunity to send satiety signals to the brain (Dhillon et al., 2016). Also, more saliva is secreted in the mouth by increasing the chewing time of food, which helps to increase fullness in the stomach. Although the effect of such a food product during oral processing on satiety is negligible, many studies have confirmed its effect on satiation (Wijlens et al., 2012).

## 6 STOMACH

Anatomically, the stomach consists of two proximal and distal parts; the proximal part includes the upper part of the stomach, including the fundus and upper part of the corpus, and the distal part includes the lower parts of the stomach, including the corpus and antrum (Janssen et al., 2011). During food intake, the proximal part of the gastric acts as a reservoir, while the distal part of the gastric mixes and breaks food into smaller pieces by a powerful and regular peristaltic contraction pattern (Goetze et al., 2009). During food intake, two critical parameters of stomach distension and gastric accommodation play an important role in satiety sensory. The gastric emptying for solid foods usually lasts between 30 and 60 min (Kwiatek et al., 2009). During this time, gastric digestion enzymes reduce the size of the solid particles and prepare them to pass through the pylorus. If the particle size is less than 1 mm, the food will leave the gastric and enter the duodenum (Hellström et al., 2006). Gastric distension is one of the most critical factors in causing satiety, and the evidence showed that gastric distension causes triggering of a stretch and tension mechanosensitive receptors, causing information to be sent to the brain via the vagus splanchnic nerves (Zhu et al., 2013). Gastric distension reduces appetite via GLP‐1 secretion and vagal afferent activation (Ohbayashi et al., 2021).

In the gastric, micronutrients and macronutrients have little effect on satiety. However, several studies have suggested that pylorus can sense the energy content of the food and allow it to enter the duodenum based on food energy (Oliver Goetze et al., 2007). However, most authors still believe that gastric distension plays the most crucial role in satiety and fullness (Cummings & Overduin, 2007). Also, one factor other than gastric distension, which plays a vital role in gastric emptying, is the product's viscosity in the distal part of the stomach. In this regard, foods producing high viscosity under digestion are less influenced by digestive enzymes. Because it is possible to reduce the gastric flow with constant force by increasing viscosity during gastric digestion, which results in a reduction of mixing of food with the gastric enzymes, these factors cause a delay in digestion of food, and as a result, gastric emptying will occur in a long time (Logan et al., 2015).

Soluble fibers are the most effective compounds in causing gastric digestion and increasing the viscosity of gastric content. The ability of soluble fiber to create high viscosity in the gastric and small intestine and the ability of fermentation in a large intestine lead to a reduction of appetite in the short term and reduction of food intake and weight loss in the long term (Efimtseva & Chelpanova, 2020). Hence, soluble fibers increase the viscosity of the gastric content by absorbing water in the gastric, causing prolonged gastric emptying. During this time, signals will be sent to the brain, and the person with a sense of fullness is less willing to eat (Emilien et al., 2018). This increase in viscosity will also continue throughout the small intestine, slowing down the absorption of nutrients. As a result, satiety hormones are released due to the absorption of nutrients into the blood, thus following slow absorption. Nutrients will remain in the blood for a long time and induce satiety for a more extended period (Fiszman & Varela, 2013; Poutanen et al., 2017).

In designing food products to regulate appetite, choosing fiber is very important. The chemical and physical structure of fibers has a significant role in water absorption. For example, cellulose is nonviscous and insoluble fiber, but ethyl hydroxyethyl cellulose and microcrystalline cellulose are soluble and viscous fibers (Chambers et al., 2015). The size of the fiber particles also plays a vital role in the amount and time required to absorb water. Also, the matrix in which the fiber is added influences a change in the structure of the fiber and its absorption rate (Salleh et al., 2019). When fibers are consumed in pure form, mixing with water or other liquids has the highest water absorption. However, the fibers present naturally in the structure of some grains, fruits, and vegetables may be influenced by the processing method, and their water absorption structure may change (Chambers et al., 2015).

## 7 SMALL INTESTINE

After digestion in the gastric, food will enter the small intestine, and nutrients will be absorbed in this stage. The small intestine acts as the most important and influential part in inducing satiety because satiety hormones will be secreted into the bloodstream at the postabsorptive stage. GLP‐1, GLP‐2, PPY, and oxyntomodulin are secreted by L distal cells of small and large intestines produced in response to low digestible or indigestible carbohydrates (Spreckley & Murphy, 2015). GLP‐1 is derived from the proglucagon molecule and is released from enteroendocrine L in distal cells, mainly in the ileum and large intestine. GLP‐1 reduces food intake and leads to a delay in gastric emptying, and this hormone remains in the blood for less than 2 min after food ingestion, which is due to rapid degradation by the enzyme dipeptidyl peptidase IV (DPP‐IV) (Barati, Javanmardi, Mousavi Jazayeri, et al., 2020; Davidson, 2009). Carbohydrates and proteins are the most critical nutrients in stimulating the secretion of GLP‐1. The difference between proteins and carbohydrates in the secretion of the GLP‐1 is that when protein is consumed, recovery time and return of hormone to the baseline level will occur earlier than carbohydrates (Suzuki et al., 2010). Eating protein and fat first and carbohydrate later effectively stimulates GLP‐1 release (Kuwata et al., 2016). A rare sugar, D‐allulose is an outstanding functional food that effectively suppresses appetite and obesity via GLP‐1 secretion and vagal afferent activation (Iwasaki et al., 2018). CCK is secreted by enteroendocrine K cells located in the duodenum and jejunum along the gastrointestinal mucosa. CCK is released by nutrients, especially proteins and fats (Ricardo‐Silgado et al., 2021).

Proteins are the most effective compounds in the secretion of satiety hormones (Barati, Javanmardi, Jabbari, et al., 2020). The rate and amount of amino acid absorption in the gastrointestinal tract determine the amount and duration of satiety. Any physicochemical changes in the structure of proteins that ultimately influence postprandial aminoacidemia patterns would alter the body's satiation patterns (Morell & Fiszman, 2017). For example, whey protein has high solubility and will be digested quickly and thoroughly. However, casein protein in the gastric will coagulate and delay digestion and leave the gastric for longer. As a result, the release of amino acids from the breakdown of casein into the blood will continue for a long time (Yang et al., 2014).

Studies have also shown that animal proteins have a more significant thermogenesis effect than plant proteins, and this thermogenesis effect causes more energy expenditure and weight loss (Chungchunlam et al., 2015). High digestibility of animal proteins compared to plant proteins can be possible. On the other hand, animal proteins all have essential amino acids, while plant proteins lack essential amino acids. It is hypothesized that the concentration of various amino acids in the blood causes the secretion of satiety hormones, such as GLP‐1, PYY, and CCK, inducing satiety and reducing appetite (Douglas et al., 2015). What kind of amino acid causes more secretion of satiety hormones? There is still no answer to this question, according to the literature. Nevertheless, the literature supports the hypothesis that animal proteins play a more significant role in reducing appetite than plant proteins because of their high digestibility and the fact that they contain essential amino acids. Therefore, the most effective proteins in reducing appetite are casein and whey proteins, playing an essential role in satiety and satiation (Pesta & Samuel, 2014; Veldhorst et al., 2008).

However, there is still a controversy over the issue of how much protein can cause satiety. Food manufacturers usually use the label “protein‐enriched” if the amount of protein is more than 20% of the total calories of the product (Blundell & Bellisle, 2013).

Protein can act as a double‐edged sword. Hence, this high amount of protein would eventually lead to overweight and obesity, which in this case, does not influence appetite. Thus, proteins can suppress appetite and, in the long‐term, cause weight loss when total energy intake is reduced, and proteins provide a more percentage of energy intake (Westerterp‐Plantenga et al., 2012). Most studies investigating the effect of protein on appetite suppression have concluded that proteins can effectively reduce appetite if 30% of energy intake is provided by proteins (Martens et al., 2013).

**FIGURE 4:**
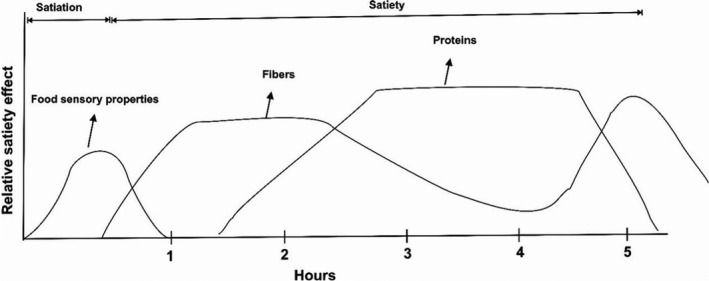
A hypothetical model of the regulation of satiation, satiety, and hunger by food ingredients

Simultaneous use of soluble fiber and protein can be a good idea to increase the duration of satiety (Figure 4) (Javanmardi et al., 2021). Fiber also increases the gastric and intestine viscosity by increasing water absorption in the gastric, reducing the effect of gastric and intestine enzymes on proteins, and digestion and absorption of food will be delayed (Adam et al., 2016). Therefore, protein absorption will occur over more extended periods. As a result, slow and continuous absorption of proteins leads to the continuous release of satiety hormones, such as GLP‐1, PYY, and CCK, into the bloodstream and causes satiety for a longer time. In digestive conditions where the gastric pH is below the isoelectric pH of the proteins, the proteins are negatively charged. Therefore, proteins’ interactions may occur between amine groups with fibers and other compounds (Zhang & Vardhanabhuti, 2014). This interaction can be in solid covalent or weak bonds, such as ionic, van der Waals, hydrogen, and hydrophobic bonds. Fibers like pectin and alginate form electrostatic bonds with amine groups of proteins during gastric digestion. This bond causes the enzyme's active site to be occupied in proteins and makes digestive enzymes unable to digest the proteins (Borreani et al., 2016; Lucy Chambers et al., 2015) entirely. Also, fibers such as glucomannan or inulin have a neutral charge and are unlikely to interact with proteins. However, because these fibers have high water absorption, they can play a vital role in increasing gastric viscosity (Mollard et al., 2014).

## 8 LARGE INTESTINE

Microorganisms of the colon play an essential role in regulating appetite. Enzymes produced by colon microorganisms cause the fermentation of dietary fibers to short‐chain fatty acids, such as acetate, propionate, and butyrate (Canfora & Blaak, 2015). Short‐chain fatty acids can bind to two receptors, free fatty acid receptors 3 and 2 (FFAR3 (GPR41) and FFAR2 (GPR43)), on the surface of bowel enteroendocrine L cells. In particular, FFAR2 acts as a marker and sensor for short‐chain fatty acids in most enteroendocrine cells, whereas FFAR3 plays this role alone in enteric neurons (Rémy Burcelin, 2017). Short‐chain fatty acids will be rapidly absorbed and metabolized in the colon. The amount of these fatty acids depends on the composition of the colon microbiota, diet, and location of the colon where fermentation takes place. It is hypothesized that short‐chain fatty acids lead to the secretion of GLP‐1. This incretin triggers insulin‐secreting cells and the food intake axis, regulating glycemia and body weight gain (Burcelin, 2010; Burcelin, 2017). In addition, some studies have reported PYY secretion due to the consumption of short‐chain fatty acids (Batterham et al., 2003). In some animal studies, fermentable carbohydrates, such as inulin (Delzenne et al., 2005), lactitol (Gee & Johnson, 2005), and fructooligosaccharide (Cani et al., 2005), increased satiety, weight loss, and increased secretion of GLP‐1 and PYY hormones. In human studies, the results have shown that fructooligosaccharides can also increase satiety and GLP‐1 concentration in the blood (Cani et al., 2006; Piche et al., 2003), but lactitol did not influence the concentration of satiety hormones (Gee & Johnson, 2005). In designing functional products, the colon can be considered the least effective area in reducing appetite. Indigestible and fermentable fibers, such as glucomannan fiber, inulin, oligofructose, methylcellulose, and beta‐glucan, can be considered one of the functional products (Wanders et al., 2011). The period of satiety will increase using these fibers in functional products to reduce appetite because the colon is the last stage, to which food will reach after passing through digestion and absorption of the gastric and intestine.

Along with fermentable fibers, probiotics can also be used to increase the efficiency of the food product in creating satiety. Studies on the effect of probiotics on satiety have been performed, in which the probiotic *Lactobacillus rhamnosus* and inulin and oligofructose have been shown to increase secretion of GLP‐1 and secretion of PYY hormones in obese people (Sanchez et al., 2017). Wouw et al. (2017) reported that the gut microbiota could influence bile acid metabolism and produce different metabolites, including short‐chain fatty acids, neuroactive, and negligible protein sequences, which can be translocated to the peripheral circulation or interact with enteroendocrine cells and the nervous system, and lead to stimulating the release of neuropeptides and peripheral hormones related to appetite and eating behavior (Van de Wouw et al., 2017). Depending on probiotic strains, doses, genetics, and microbial constitutions, probiotics have a different effect on appetite (Cabral et al., 2020). Therefore, probiotics and prebiotics in functional foods can prolong satiety time by claiming to reduce appetite.

## 9 CONCLUSION

In general, it can be concluded that an accurate choice of ingredients for functional food products claiming satiety induction and the effect of these compounds on the body has a significant role in designing products with the most excellent satiety effect. Therefore, it is possible to design a product that can create maximum satiety and maintain satiety's effect for a longer time by considering target areas in the mouth, gastric, small and large intestines. Soluble and fermentable fibers with the different effect in the mouth, gastric, and large intestines can be considered the most efficient compound in a functional product claiming to reduce appetite. Besides, proteins have the highest ability to induce the secretion of satiety hormones after absorption in the small intestine. In addition, sensory properties of the food product, such as odor, taste, and texture, can also be regarded as factors enhancing the satiety effect.

## CONFLICTS OF INTEREST

The authors declare no conflict of interest.

## AUTHOR CONTRIBUTION

**Mina Esmaeili:** conceptualization (equal) ; dataCuration (equal); formalAnalysis (equal); fundingAcquisition (equal); investigation (equal); methodology (equal); projectAdministration (equal); resources (equal); software (equal); supervision (equal); validation (equal); visualization (equal); writingOriginalDraft (equal); writingReviewEditing (equal). **Marjan Ajami:** conceptualization (equal) ; dataCuration (equal); formalAnalysis (equal); fundingAcquisition (equal); investigation (equal); methodology (equal); projectAdministration (equal); resources (equal); software (equal); supervision (equal); validation (equal); visualization (equal); writingOriginalDraft (equal); writingReviewEditing (equal). **Meisam Barati:** conceptualization (equal) ; dataCuration (equal); formalAnalysis (equal); fundingAcquisition (equal); investigation (equal); methodology (equal); projectAdministration (equal); resources (equal); software (equal); supervision (equal); validation (equal); visualization (equal); writingOriginalDraft (equal); writingReviewEditing (equal). **Fardin Javanmardi:** conceptualization (equal) ; dataCuration (equal); formalAnalysis (equal); fundingAcquisition (equal); investigation (equal); methodology (equal); projectAdministration (equal); resources (equal); software (equal); supervision (equal); validation (equal); visualization (equal); writingOriginalDraft (equal); writingReviewEditing (equal). **Anahita Houshiarrad:** conceptualization (equal) ; dataCuration (equal); formalAnalysis (equal); fundingAcquisition (equal); investigation (equal); methodology (equal); projectAdministration (equal); resources (equal); software (equal); supervision (equal); validation (equal); visualization (equal); writingOriginalDraft (equal); writingReviewEditing (equal). **Amin Mousavi Khaneghah:** conceptualization (equal) ; dataCuration (equal); formalAnalysis (equal); fundingAcquisition (equal); investigation (equal); methodology (equal); projectAdministration (equal); resources (equal); software (equal); supervision (equal); validation (equal); visualization (equal); writingOriginalDraft (equal); writingReviewEditing (equal).

## CONSENT FOR PUBLICATION

Not applicable.

## ETHICS APPROVAL AND CONSENT TO PARTICIPATE

Ethical approval was not necessary for the current study, as no new participants were recruited for the research.

## Data Availability

Not applicable.
